# Effects of Vitamin D Supplementation and Seasonality on Circulating Cytokines in Adolescents: Analysis of Data From a Feasibility Trial in Mongolia

**DOI:** 10.3389/fnut.2019.00166

**Published:** 2019-10-23

**Authors:** Sergey Yegorov, Sabri Bromage, Ninjin Boldbaatar, Davaasambuu Ganmaa

**Affiliations:** ^1^Department of Pedagogical Mathematics and Natural Science, Faculty of Education and Humanities, Suleyman Demirel University, Almaty, Kazakhstan; ^2^Department of Biology, School of Science and Humanities, Nazarbayev University, Nur-Sultan, Kazakhstan; ^3^Department of Nutrition, Harvard T.H. Chan School of Public Health, Boston, MA, United States; ^4^Department of Radiation Oncology, Dana-Farber Cancer Institute, Brigham and Women's Hospital, Boston, MA, United States; ^5^Channing Division Network of Medicine, Brigham and Women's Hospital, Harvard Medical School, Boston, MA, United States

**Keywords:** vitamin D deficiency, cholecalciferol supplementation, cytokines, chemokines, Mongolia, Northeast Asia, adolescents

## Abstract

Vitamin D deficiency is prevalent in human populations and has been linked to immune dysfunction. Here we explored the effects of cholecalciferol supplementation on circulating cytokines in severely vitamin D deficient [blood 25(OH)D << 30 nmol/L] adolescents aged 12–15 from Mongolia. The study included 28 children receiving 800 IU daily cholecalciferol for 6 months spanning winter and spring, and 30 children receiving placebo during the same period. The levels of 25(OH)D were assessed at baseline, 3 and 6 months. Twenty-one cytokines were measured in serum at baseline and at 6 months. Changes in 25(OH)D and cytokines were assessed using paired parametric tests. The median blood 25(OH)D concentration at baseline was 13.7 nmol/L (IQR = 10.0–21.7). Supplementation tripled blood 25(OH)D levels (*p* < 0.001) and was associated with elevated interleukin (IL)-6 (*p* = 0.043). The placebo group had reduced macrophage inflammatory protein (MIP)-1α (*p* = 0.007) and IL-8 (*p* = 0.034) at 6 months. Although limited by a small sample size, these findings suggest that cholecalciferol supplementation and seasonality may impact systemic immunity in adolescents, identifying chemokines as potentially important biomarkers of vitamin D status in this Northeast Asian population. Larger clinical trials are warranted to validate these results.

**Clinical Trial Registration:** www.ClinicalTrial.org, Identifier: NCT01244204.

## Introduction

Accumulating evidence indicates that vitamin D has important non-skeletal functions, particularly in the immune system (1–5). Thus, vitamin D deficiency has been associated with increased risk for diseases tightly linked to immune function, such as autoimmune conditions and respiratory tract infections (1, 4–6). Notably, a recent meta-analysis found that vitamin D supplementation significantly reduced the risk of acute respiratory infections most prominently in individuals with low serum 25(OH)D concentrations (<25 nmol/L) (7). In addition, our earlier studies in Mongolian children, in whom vitamin D deficiency [25(OH)D <25 nmol/L or 10 ng/ml (8)] prevalence exceeds 80%, indicated a significant reduction of acute respiratory tract infection associated with cholecalciferol supplementation (9, 10). Similar observations were made regarding the relationship of vitamin D and immunity to TB infection in other cohorts (2).

The *in vivo* effects of vitamin D on immunity are still incompletely understood in the setting of different human populations and genetic backgrounds (3), although studies performed *in vitro* and in animal models indicate that the nature of vitamin D effects on immunity is context- and cell type-dependent. For example, vitamin D exerts stimulatory effects on monocytes and macrophages inducing interleukin (IL)-1 production, while modulating adaptive immune responses by increasing IL-10 production by dendritic and T cells (1, 5) and these effects are differentially regulated in the presence of pathogen stimuli (11).

Given the protective effects of vitamin D against respiratory infections and the evidence of the vitamin's immune roles *in vitro*, here we tested the hypothesis that 6 months of cholecalciferol supplementation in severely vitamin D-deficient adolescents would result in a significant change in the circulating mediators of antiviral and antibacterial immunity.

## Methods

### Study Setting and Participant Recruitment

This study is part of a feasibility pilot trial (completed in 2010) to assess the effects of vitamin D supplementation on latent TB incidence in 120 Mongolian children (ClinicalTrial.org ID: NCT01244204) (9). The screening and recruitment of participants was described previously (9, 12). Briefly, children aged 12–15 years residing in Ulaanbaatar, the capital of Mongolia, were recruited. The study intervention consisted of 800 IU vitamin D or placebo (Tishcon Corp., Salisbury, MD) daily for 6 months from November 2009 to May 2010, during the coldest period of the Mongolian year. The choice of the vitamin D supplementation regimen was guided by the National Academy of Medicine (formerly the Institute of Medicine) (13) and the Endocrine Society (14) guidelines, according to which the recommended dietary allowance for vitamin D in adolescents ranges from 600 to 1000 IU/day.

For the current analysis, 58 paired serum samples (baseline and 6 months) were randomly selected from the parent placebo (*n* = 30/59) and supplemented (*n* = 28/61) groups. This study was designed to supply pilot data for future larger studies in the same cohort, therefore no formal sample size calculations were performed, and sample size was determined based on the available study budget. All research procedures were approved by the institutional review boards of the Mongolian Ministry of Health, National University of Mongolia and the Harvard School of Public Health (Ref. #16571). Written consent to participate was collected from both children and their parents.

### Sample Collection and Diagnostic Testing

Blood (8.0 ml) was collected by venipuncture into red top tubes (Becton Dickinson). Serum was isolated by centrifugation and stored at −80°C prior to analysis. Measurement of 25(OH)D was performed using LIAISON 25-OH Vitamin D TOTAL assay [DiaSorin S.p.A. (Italy)] at 0, 3, and 6 months. The concentrations of 21 cytokines representing chemokines (macrophage inflammatory protein (MIP)-1α, MIP-1β, MIP-3α, interleukin (IL)-8, fractalkine, interferon-inducible T-cell alpha chemoattractant (ITAC), homeostatic (IL-2, IL-7, granulocyte-macrophage colony-stimulating factor (GM-CSF), classic proinflammatory (IL-1β, IL-6, tumor necrosis factor (TNF), Th17-type proinflammatory (IL-17α, IL-23), regulatory (IL-10, IL-21) as well as Type I [interferon (IFN)γ, IL-12] and Type II cytokines (IL-13, IL-4, IL-5) (see Supplementary Table 1 for details on the cytokines and assay sensitivity) were measured at baseline and at 6 months using the Human High Sensitivity T Cell Panel (HSTCMAG-28SK) on a MAGPIX instrument (EMD Millipore). All experimental assays were performed by research personnel blinded to the supplementation status of participants.

### Statistical Analysis

Differences in demographic characteristics between the cholecalciferol supplemented and placebo groups were assessed using Independent-Samples Mann-Whitney *U* and Chi-Square Tests. Mean height-for-age and height-for-BMI z scores were calculated using WHO Anthro software (15). Cytokine concentrations were log10-transformed prior to analysis to facilitate visualization, while hypothesis testing was performed on the original (untransformed) data. To enhance the power of analysis, serum 25(OH)D and cytokine concentrations across study visits were compared using paired *t*-test. Differences between the placebo and cholecalciferol supplemented groups at each time point were assessed by one sample *t*-test. To compare the cumulative direction change in cytokine concentrations between the supplemented and placebo groups, we first calculated the proportion of cytokines that were found on average increased or decreased at follow-up for each participant group (solid dots for each cytokine in **Figure 2**), and then performed a Chi-Square test of the null hypothesis that there was no significant change in cumulative cytokine concentrations between the placebo and cholecalciferol-supplemented children. All statistical analyses and graphing were performed using IBM SPSS V.23 (NY, US) and GraphPad Prism V.6.0. (CA, US).

## Results

### Participant Demographics

Samples from a total of 58 children were analyzed. At baseline the median serum 25(OH)D concentration was 13.7 nmol/L and basic socio-demographic characteristics were not significantly different between the supplemented and placebo groups (Supplementary Table 2).

### Changes in Systemic 25(OH)D Concentrations

**Figure 1:**
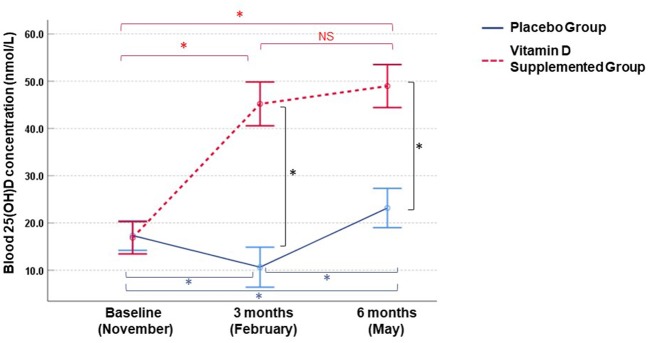
Longitudinal changes in blood 25(OH)D concentrations in Mongolian children. Measurements were performed in vitamin D3 supplemented (*n* = 25) and placebo (*n* = 30) groups. Circles and bars denote means and 95% confidence intervals, respectively. Intraindividual changes within each group and inter-group differences were assessed by paired *t*-test and one-sample *t* test (*p* < 0.05), respectively. *p-values <0.001, NS, not significant.

First, we examined blood 25(OH)D levels at baseline (November) and 3 and 6 months (February and May, respectively) (Figure 1). Children receiving placebo had significantly reduced 25(OH)D in February (mean = 10.6 nmol/L, mean fold change (MFC) = 1.4, *p* < 0.001) and an increase of 25(OH)D in May (mean = 23.2 nmol/L, MFC = 1.5, *p* < 0.001) compared to November. On the other hand, children receiving cholecalciferol exhibited an elevation of 25(OH)D in February (mean = 44.3 nmol/L, MFC = 3.1, *p* < 0.001) and this increase was sustained in May (mean = 49.0 nmol/L, MFC = 3.5, *p* < 0.001) compared to November. Compared to the placebo group, cholecalciferol supplemented participants had significantly higher blood 25(OH)D levels in both February (4.2-fold, *p* < 0.001) and May (2.1-fold, *p* < 0.001) (Figure 1).

### Changes in Systemic Cytokine Concentrations

**Figure 2:**
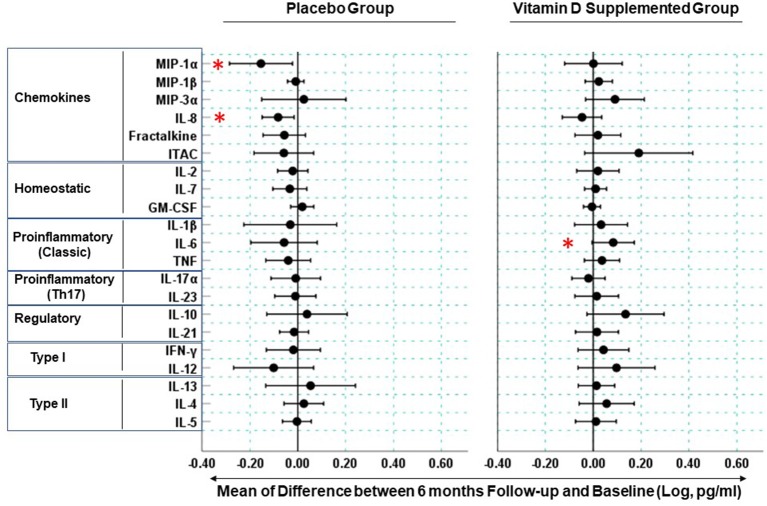
Longitudinal changes in blood cytokine concentrations in Mongolian children. Measurements were performed on serum samples from vitamin D supplemented (*n* = 25) and placebo (*n* = 27) groups. Circles represent means of difference between log-transformed cytokine concentrations at the 6 month follow-up visit and baseline. Bars are 95% confidence intervals. Cytokine change is considered significant when its respective confidence intervals are found entirely on the positive or negative sides of the x-axis and are not spanning the “0” reference line. Stars denote the cytokines (MIP-1α, IL-8, IL-6) exhibiting statistically significant changes (*p* < 0.05) see Supplementary Figure 1 for participant-level data for these cytokines.

Due to missing data, paired cytokine analysis was performed for 52 out of 58 (27 placebo and 25 supplemented) participants. The mean cytokine concentrations in the placebo and supplemented groups at baseline and 6 months are listed in Supplementary Table 3; a cross-sectional analysis did not detect any significant differences (at *p* ≤ 0.05) in the cytokine concentrations between the placebo and supplemented groups at baseline or at 6 months. Therefore, we compared intra-participant changes in cytokine concentrations between baseline and 6 months after study initiation (Figure 2). In the supplemented group, 85.7% (18/21) and 14.3% (3/21) of measured cytokines exhibited a positive and negative change, respectively, while in the placebo group the mean difference in cytokine concentrations was negative for 76.2% (16/21) and positive for 23.8% (5/21) of the assessed cytokines. Thus, cholecalciferol supplementation had a significant effect on the cumulative direction of change in blood cytokine concentrations compared to placebo (Chi-square statistic = 16.2, *p* < 0.001). The cholecalciferol supplemented group had elevated IL-6 (MFC = 1.4, *p* = 0.043) (Figure 2 and Supplementary Figure 1), while the levels of chemokines MIP-1α and IL-8 were significantly reduced in the placebo group (MFC = 2.38, *p* = 0.007 and MFC = 1.31, *p* = 0.034 for MIP-1α and IL-8, respectively, Figure 2 and Supplementary Figure 1).

## Discussion

Here we longitudinally examined the effect of vitamin D supplementation on the circulating cytokines representing several different immune pathways. We conducted the study in Mongolia, where extreme vitamin D deficiency is common (9, 12) and vitamin D supplementation results in a substantial increase of circulating 25(OH)D levels (9, 12, 16). Here the supplemented group's 25(OH)D levels increased gradually from 13.7 to 49.0 nmol/L, while the un-supplemented adolescents remained severely deficient with blood 25(OH)D levels remaining <25 nmol/L throughout the study. Interestingly, in May the placebo group had higher 25(OH)D concentrations compared to November likely due to higher sun exposure in Spring, although these levels were still under the “critical” threshold (7) of 25 nmol/L (Figure 1).

Our findings should be interpreted in the light of several limitations. First, the study was designed as a pilot with a small sample size, which reduced the power to detect small differences in blood cytokine concentrations due to high inter-individual variability (see Supplementary Table 3). However, we addressed this limitation by performing a paired statistical analysis and compared changes in cytokine concentrations intra-individually, allowing us to reduce the effects of inter-individual variability. The small sample size also limited our choices for data analysis, and we were unable to employ multivariable modeling in this study. The cytokine panel size, time points covered by the study and a rather conservative vitamin D regimen are other important limitations of this work. Nevertheless, this study provides novel hypothesis-generating insights at the impact of vitamin D supplementation on the immunology of severely vitamin D deficient Mongolian adolescents, as discussed below.

The effects of vitamin D supplementation on human immunology have now been assessed by several studies; including large scale randomized controlled trials and rigorous meta-analyses, which reported a lack of any effect of vitamin D supplementation on systemic biomarkers, such as IL-6 or C-reactive protein (17–19). Notably, most of these studies describe data from Western countries and there is a paucity of similar research from lower resource settings, such as Mongolia. To the best of our knowledge, our study is the first to report on the effects of supplementing vitamin D on systemic immunology in a Mongolian cohort, one distinguishing feature of which is the severity of vitamin D deficiency, where the mean baseline/pre-supplementation level of circulating vitamin D is 13.7 nmol/L and hence a few-fold lower compared to that seen in many other studies from Western countries (17, 18). This is important since a recent meta-analysis indicates that 25 nmol/L is a critical threshold of vitamin D deficiency associated with elevated risk for acute respiratory infection (7) and other studies demonstrate that vitamin D supplementation alters circulating IFN-g and IL-10 levels in individuals with vitamin D insufficiency (20), suggesting that vitamin D supplementation exerts most effect on human immunity in the context of severe vitamin D deficiency.

Compared to baseline, 6 months of cholecalciferol supplementation resulted in a cumulative shift toward elevation for a majority (~86%) of measured cytokines. This was in stark contrast with the effect seen in the placebo group, where compared to baseline ~76% of cytokines exhibited a cumulative shift toward reduction, suggesting that vitamin D deficiency could contribute to a gradual change of immune variables over winter. Further, the vitamin D-deficient children had significantly reduced MIP-1α (CCL3) and IL-8 (CXCL8) concentrations at 6 months compared to baseline. This effect was not seen in the supplemented participants, suggesting that vitamin D deficiency is implicated in the reduction of these chemokines playing roles in leukocyte homing and activation and mediating antibacterial and antiviral immune responses (21, 22). Previously, vitamin D supplementation in the context of chronic kidney disease was implicated in decreasing the concentrations of another chemokine, monocyte chemoattractant protein-1, and our findings provide further evidence for the role of vitamin D in chemokine homeostasis (23).

The increased IL-6 observed in the supplemented children is consistent with IL-6 up-regulation in the peripheral blood mononuclear cells of vitamin D supplemented multiple sclerosis patients (24). At the same time vitamin D deficiency was associated with high IL-6 (25) and vitamin D supplementation down-regulated IL-6 in some studies (26–29). These seemingly contradictory findings regarding IL-6 and vitamin D could be attributed to various confounders and differences among the studies, such as the duration and dosage of vitamin D supplementation, the participants' genetic background, underlying clinical conditions, effects of clinical therapy, and extent of vitamin D insufficiency/deficiency at baseline.

Our findings are consistent with other studies reporting an increase of IL-10 in vitamin D supplemented individuals (20, 30, 31), as we also observed a strong trend to elevated IL-10 in the supplemented children. Somewhat surprisingly, we saw no change in IFN-γ in our study, the Type I cytokine that was significantly elevated in vitamin D-supplemented US and Mexican adults (20, 30), which could reflect the diverse effects of vitamin D in different age and/or ethnic groups.

An important factor to consider when interpreting our results in the light of earlier research is participant age, which has important implications for the assessment of immune responses (32). A recent study by Berlanga-Taylor et al. (19), for example, reported a lack of any effect of 1 year-long vitamin D supplementation on circulating IFN-γ, IL-10, IL-8, IL-6, or TNF. However, the mean age of participants in this study was 72 years and their mean pre-supplementation vitamin D level was 50.0 nmol/L, thus precluding a direct comparison of this study's outcomes with our results.

In summary, this study for the first time assessed the immune effects of cholecalciferol supplementation in severely vitamin D deficient adolescents from Northeast Asia. Although it is challenging to differentiate between the effects of vitamin D supplementation versus seasonality, cholecalciferol supplementation appeared to influence systemic immune mediators in this population. The small sample size precludes making strong conclusions based on the study findings. A larger clinical trial is warranted to validate these results and explore in detail the utility of the identified cytokines as biomarkers of vitamin D-mediated immune (dys-) function as well as the associations of the relevant immune pathways with clinical outcomes.

## Data Availability Statement

All data generated or analyzed during this study are included in this published article. The authors acknowledge that an earlier version of this work was uploaded to medRxiv, a preprint server for health sciences, and is publicly available at: https://www.medrxiv.org/content/10.1101/19001842v1.

## Ethics Statement

The studies involving human participants were reviewed and approved by Harvard School of Public Health IRB and Mongolian Ministry of Health ERB. Written informed consent to participate in this study was provided by the participants' legal guardian/next of kin.

## Author Contributions

SY and DG: conceptualization. SY and SB: formal analysis. SY and NB: methodology. DG: funding acquisition, supervision, project administration, and resources. SY: visualization and writing—original draft. All co-authors: writing—review and editing.

### Conflict of Interest

The authors declare that the research was conducted in the absence of any commercial or financial relationships that could be construed as a potential conflict of interest.
