# Supplementary material for: Effects of Vitamin D Supplementation and Seasonality on Circulating Cytokines in Adolescents: Analysis of Data From a Feasibility Trial in Mongolia
Source: Front Nutr. 2019 Oct 23;6:166. doi: 10.3389/fnut.2019.00166 (PMC6819500; doi:10.3389/fnut.2019.00166)
Supplement: Supplementary file 1 [file Data_Sheet_1.PDF]

Effects of vitamin D supplementation and seasonality on circulating cytokines in adolescents: analysis of data from a feasibility trial in Mongolia.

Sergey Yegorov[1,2]*, Sabri Bromage[3], Ninjin Boldbaatar[4], Davaasambuu Ganmaa [3,5]

[1] Department of Science Education, Faculty of Education and Humanities, Suleyman Demirel University, Almaty, Kazakhstan

[2] School of Science and Technology, Nazarbayev University, Astana, Kazakhstan

[3] Department of Nutrition, Harvard T.H. Chan School of Public Health, Boston, MA, United States of America

[4] Department of Radiation Oncology, Dana-Farber Cancer Institute, Brigham and Women's Hospital, Boston, MA, United States of America

[5] Channing Division Network of Medicine, Brigham and Women's Hospital, Harvard Medical School, Boston, MA, United States of America

Supplementary Table 1. Description of the cytokines assessed by multiplex ELISA in the serum of Mongolian adolescents.

#	Cytokine	Category	Full name	Alternative name	Lowest limit of detection (pg/mL)
1	**MIP-1α**	Chemokine	Macrophage inflammatory protein 1α	CCL3	0.93
2	**MIP-1β**	Chemokine	Macrophage inflammatory protein 1β	CCL4	0.69
3	**MIP-3 α**	Chemokine	Macrophage inflammatory protein 3α	CCL20	0.79
4	**IL8**	Chemokine	Interleukin-8		0.12
5	**Fractalkine**	Chemokine	Fractalkine	CX3CL1	7.75
6	**ITAC**	Chemokine	Interferon-inducible T-cell alpha chemoattractant	CXCL11/IP-9	1.24
7	**IL-2**	Homeostatic	Interleukin-2		0.18
8	**IL-7**	Homeostatic	Interleukin-7		0.43
9	**GM-CSF**	Homeostatic/Proinflam matory	Granulocyte-macrophage colony-stimulating factor		0.33
10	**IL-1β**	Pro-inflammatory	Interleukin-1β		0.14
11	**IL-6**	Pro-inflammatory	Interleukin-6		0.11
12	**TNF**	Pro-inflammatory	Tumour necrosis factor		0.16
13	**IL-17A**	Pro-inflammatory (Th17)	Interleukin-17A		0.31
14	**IL-23**	Pro-inflammatory (Th17)	Interleukin-23		3.06
15	**IL-10**	Regulatory	Interleukin-10		0.51
16	**IL-21**	Regulatory	Interleukin-21		0.14
17	**IFN-γ**	Type 1	Interferon-gamma		0.47
18	**IL-12p70**	Type 1	Interleukin-12		0.16
19	**IL-13**	Type 2	Interleukin-13		0.24
20	**IL-4**	Type 2	Interleukin-4		1.07
21	**IL-5**	Type 2	Interleukin-5		0.1

Supplementary Table 2. Baseline Characteristics of Children in the Vitamin D Supplementation and Placebo groups.[1]

Participant characteristic	Entire cohort (n=58)	Placebo group (n=30)	Supplemented group (n=28)	p value[1]
**Median age (IQR)**	14.0 (13.0-15.0)	13.0 (13.0-15.0)	14.0 (13.0-15.0)	0.899
**Male sex, n (%)**	28 (48)	15 (50)	13 (46)	0.786
**Housing, living in a ger*, n (%)**	30 (52)	18 (60)	12 (42)	0.192
**Consuming vitamins or minerals outside the study, n (%)**	27 (47)	16 (53)	11 (39)	0.284
**Mean height-for-age z score (CI)**	-0.66 (-0.949/-0.360)	-0.46 (-0.868/-0.059)	-0.85 (-1.30/-0.410)	0.188
**Mean height-for-BMI z score (CI)**	-.0163 (-0.246/0.214)	0.06 (-0.266/-0.380)	-0.09 (-0.439/0.255)	0.521
**Mean N of people residing with (CI)**	5.07 (4.66-5.47)	5.37 (4.72-6.01)	4.75 (4.26-5.24)	0.129
**Median serum 25(OH)D level, nmol/L (IQR)**	13.6 (10.0-21.7)	13.6 (10.0-22.9)	13.7 (10.0-20.7)	0.762

[1] Differences between the supplemented and placebo groups were assessed using Independent-Samples Mann-Whitney U and Chi-Square Tests. None of the variables were significantly different between the vitamin D supplemented and placebo groups at baseline.

BMI= body mass index, CI= confidence interval

*Mongolian tent-like dwelling, yurt.

Supplementary Table 3. Mean serum concentrations (in pg/ml) of cytokines in the Vitamin D Supplementation and Placebo groups.

	Placebo group (n=27)		Vitamin D supplemented group (n=25)		P value*
	Baseline	Six months	Baseline	Six months	
Cytokine	Mean (SD)	Mean (SD)	Mean (SD)	Mean (SD)	
MIP-1α	13.6 (5.5)	11 (4.7)	12.7 (8.8)	11 (5.3)	0.825
MIP-1β	20.6 (8.3)	20.3 (7.9)	20.7 (17)	20.5 (13.3)	0.840
MIP-3α	25 (12.3)	27.1 (14.9)	22.6 (12.6)	24.6 (11.7)	0.748
IL8	7.5 (5.1)	5.9 (3.1)	6.6 (4.1)	5.6 (3)	0.954
Fractalkine	99.9 (51.8)	99.1 (59.2)	101.2 (65.7)	99.3 (58)	0.681
ITAC	35.8 (52.2)	31.3 (42.6)	33.3 (66.9)	48.9 (98.5)	0.342
IL-2	4 (2.5)	3.9 (2.3)	3.9 (2.2)	3.8 (2.2)	0.913
IL-7	14 (7.3)	14.1 (8.6)	13.8 (7)	13.6 (6.5)	0.995
GM-CSF	97.6 (54.9)	101.3 (60)	113.1 (61.1)	109.8 (58.6)	0.631
IL-1β	5.4 (3.2)	5.2 (2.9)	5 (2.9)	4.9 (2.8)	0.965
IL-6	2 (1.2)	2 (1.5)	1.5 (0.8)	1.7 (0.9)	0.591
TNF	12 (4.8)	11.6 (4.8)	10.8 (5)	11.1 (4.2)	0.799
IL-17a	10.4 (8)	10.7 (7.5)	10 (5.3)	9.6 (5.8)	0.555
IL-23	212.5 (195.2)	222.5 (263.2)	252.2 (250.6)	231.9 (222.6)	0769
IL-10	9.8 (6.2)	9.4 (5.6)	7.9 (4)	10.1 (6.4)	0.613
IL-21	3.4 (2.8)	3.3 (2.8)	2.7 (1.4)	2.7 (1.3)	0.339
IFN-γ	27.9 (14.7)	27.1 (13.9)	26.8 (14.9)	25.6 (10.4)	0.741
IL-12	4.8 (4.3)	4.8 (4.2)	3.7 (2.2)	4 (2.6)	0.457
IL-13	5.3 (3)	5.2 (2.9)	4.5 (2.5)	4.5 (2.3)	0.344
IL-4	35.1 (26.3)	39 (28.2)	28.1 (18.4)	29.2 (18.7)	0.206
IL-5	4.8 (2.5)	4.9 (2.5)	4.3 (2.4)	4.2 (2.1)	0.387

SD: standard deviation; * p value given for independent samples t-test comparing the placebo and supplemented groups at six months.

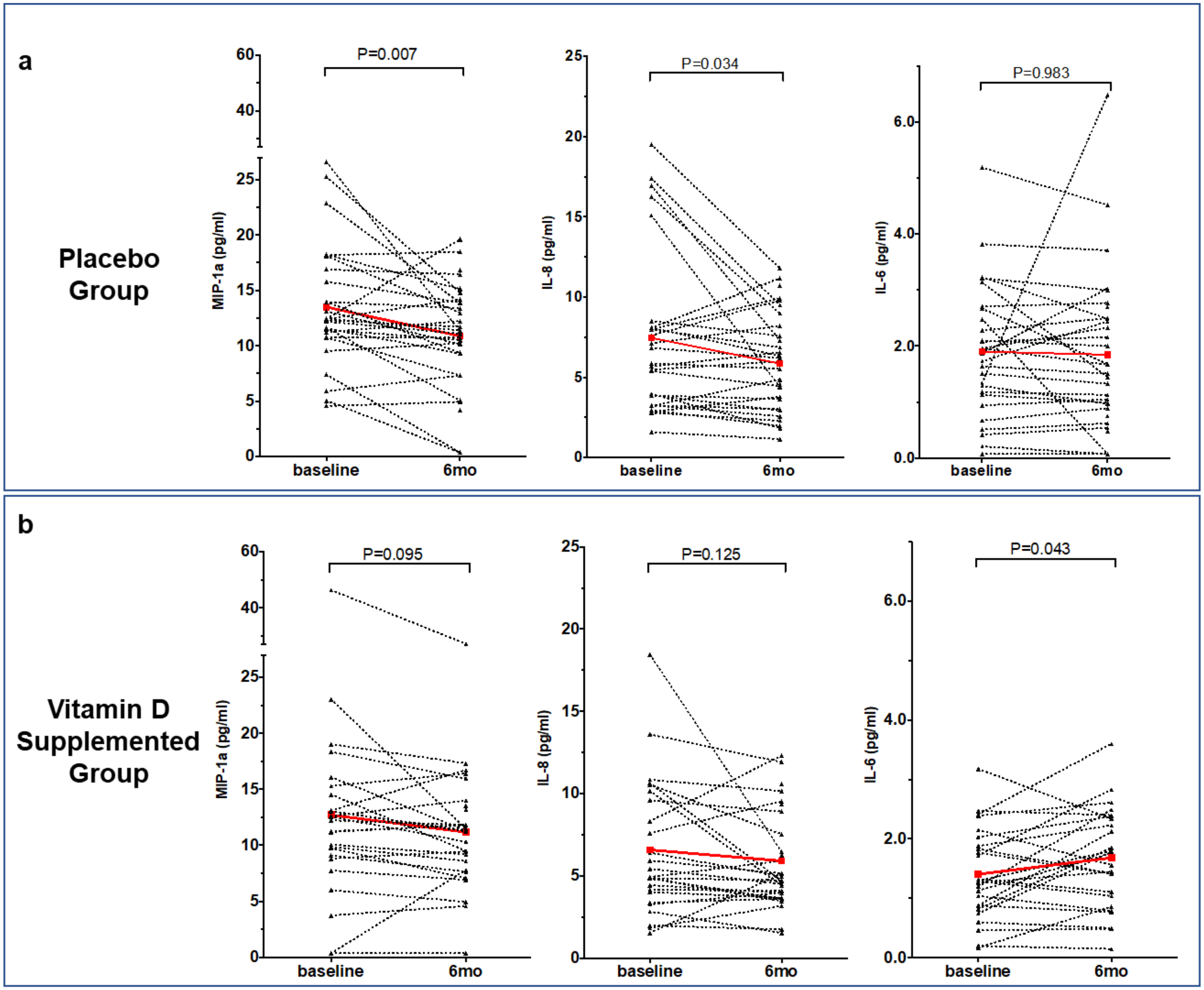

Supplementary Figure 1. Longitudinal changes in blood MIP-1α, IL-8 and IL-6 observed in Mongolian children. Participant-level data are depicted by dotted lines, red dots indicated mean level at each visit; n=27 and 25 for the placebo and supplemented groups, respectively.
